# Determining the N-terminal orientations of recombinant transmembrane proteins in the *Escherichia coli* plasma membrane

**DOI:** 10.1038/srep15086

**Published:** 2015-10-14

**Authors:** Chien-Hsien Lee, Chia-Cheng Chou, Min-Feng Hsu, Andrew H.-J. Wang

**Affiliations:** 1 The Institute of Biological Chemistry, Academia Sinica, Taipei, Taiwan; 2 Core Facilities for Protein Structural Analysis, Academia Sinica, Taipei, Taiwan

## Abstract

*In silico* algorithms have been the common approach for transmembrane (TM) protein topology prediction. However, computational tools may produce questionable results and experimental validation has proven difficult. Although biochemical strategies are available to determine the C-terminal orientation of TM proteins, experimental strategies to determine the N-terminal orientation are still limited but needed because the N-terminal end is essential for membrane targeting. Here, we describe a new and easy method to effectively determine the N-terminal orientation of the target TM proteins in *Escherichia coli* plasma membrane environment. D94N, the mutant of bacteriorhodopsin from *Haloarcula marismortui*, can be a fusion partner to increase the production of the target TM proteins if their N-termini are in cytoplasm (N_in_ orientation). To create a suitable linker for orientating the target TM proteins with the periplasmic N-termini (N_out_ orientation) correctly, we designed a three-TM-helix linker fused at the C-terminus of D94N fusion partner (termed D94N-3TM) and found that D94N-3TM can specifically improve the production of the N_out_ target TM proteins. In conclusion, D94N and D94N-3TM fusion partners can be applied to determine the N-terminal end of the target TM proteins oriented either N_in_ or N_out_ by evaluating the net expression of the fusion proteins.

Transmembrane (TM) proteins are essential for cell function and viability^1^. However, they must be correctly embedded in the appropriate membrane for proper folding, activity, and stability. The genes encoding these proteins, e.g., transporters, receptors, and channels, comprise ~20–30% of all coding sequences in a typical genome^1^,^2^. Moreover, membrane proteins currently represent ~50% of the drug targets^3^.

How TM proteins generate their correct topologies and how they fold into functional structures in membrane environments are important questions needing answers if we are to understand TM protein biogenesis^4^,^5^. For polytopic α-helical TM proteins, their topologies can be defined by the number of helices and how these helices are oriented in the lipid bilayer, i.e., with their termini positioned in the cytosol (in) or on the opposite side of the membrane (out). However, orientation information on TM proteins is lost during X-ray crystallographic studies because the proteins are first extracted from the membrane and solubilized in an isotopic medium, i.e., a detergent. To predict the topology and structure of TM proteins *in vivo*, computational tools are available^4^,^6^,^7^, and some experimental biochemical methods exist^8^,^9^. However, *in silico* simulation and prediction studies still require experimental validation, a task that has proven difficult to date^10^,^11^. Because TM protein topology depends on multiple determinants, and, in certain cases, insufficient data are considered in predictive algorithms resulting in misaligned and/or misoriented helices^4^,^7^. In addition, only a few TM protein structures have been deposited into the Protein Database that might be used for *in silico* comparisons and statistical analyses (http://blanco.biomol.uci.edu/mpstruc/)^12^. Moreover, although some soluble protein tags, e.g. β-lactamase (BlaM) and green fluorescent protein (GFP), can be used to determine the orientation of C-terminal TM protein residues^8^,^9^,^13^, methods for determining the orientation of the N-terminus are limited.

During TM protein biogenesis in *Escherichia coli*, usually the N-terminal region is the first to be inserted into the plasma membrane via a coordinated effort involving the ribosome-nascent polypeptide complex and the translocon (reviewed in Luirink *et al.*^14^) and, therefore, is critical for both this process and the proper orientation of a protein in the membrane^4^,^9^,^15^. Mutation or modification of the N-terminus of a TM protein may have a negative effect on membrane targeting and the native state of the protein^9^,^16^. Consequently, only limited experimental method exists to assess the N-terminus location of a mature TM protein. To address this deficiency, we have developed a quick and easy method to obtain N-terminal topological information concerning the recombinant TM proteins inserted into the *E. coli* plasma membrane.

Previously, we developed an expression vector for TM proteins^17^ that encodes a mutant (D94 → N) of the TM protein bacteriorhodopsin from *Haloarcula marismortui* (HmBRI/D94N, denoted hereafter as D94N) with a cytoplasm-oriented C-terminus (C_in_) when embedded in a membrane. This design increases the expression of a downstream targeted TM protein with the same N-terminus orientation (N_in_). However, this design is not suitable for N_out_ targets. We surmised that by adding a linker with odd number TM helices between D94N and the N_out_ targets will serve the purpose.

In a separate project in our laboratory, we have designed computationally a four-helical bundle membrane protein (called 4TM) derived from the soluble cytochrome b_562_. We tested this newly designed 4TM and its subsystems of 1TM, 2TM and 3TM as possible linkers. We found that 3TM is the most effective one. By coupling D94N with 3TM, we created a D94N-related TM protein construct with a C-terminus exposed to the *E. coli* periplasmic face (C_out_) which improves the expression of N_out_ TM proteins. Therefore, using our two fusion partners, i.e., D94N and D94N-3TM, the N-terminal orientation of the target TM protein can be deduced efficiently by assessing the net expression of the protein product. Fusing the target TM protein downstream to the compatible fusion partner can produce a correct topology, whereas fusing the target TM protein to the incompatible one might results in a unstable protein due to the interrupted topology. The unstable TM proteins are presumably proteolytically removed by *in vivo* quality control system^18^,^19^.

We assessed our method using three TM proteins with known N_out_ orientations, i.e., NADH dehydrogenase subunit A (NuoA) and subunit J (NuoJ)^20^, nitrate reductase 1 gamma chain (NarI)^21^, and another one with a proposed N_in_ orientation, undecaprenyl pyrophosphate phosphatase (UppP)^22^ from *E. coli*. The results are consistent with our expectations. We next used our method to determine the orientations of the N-termini of seven additional TM proteins to assess the practicality of this strategy. Our method is quick and easy to perform and does not rely on mutagenesis, structural determination, or chemical labeling to determine the topology of the N-terminal residue in a TM protein. By combining the protein tags for C-terminal orientation determination with D94N-related fusion partners, a more comprehensive topological information of a target TM protein can be obtained.

## Results

### Computational design of a TM protein

**Figure 1:**
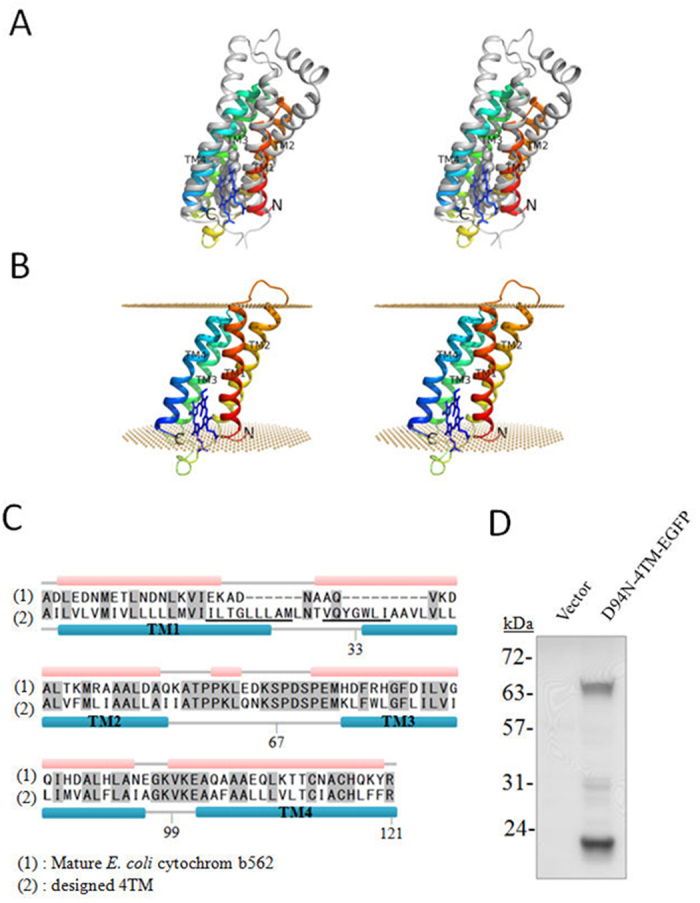
The amino acid sequences of cytochrome b_562_ and 4TM. The wall-eye stereo view of (**A**) the superimposed structures of cytochrome b562 (PDB: 3DE9, rainbow) and TM1-TM4 of cytochrome b from cytochrome bc1 complex (PDB: 3H1J, chain C, gray), and (**B**) the structure model of membrane-embedded 4TM showed in rainbow. The heme group in cytochrome b is presented as spheres and ball-and-stick in cytochrome b_562_. (**C**) The amino acid sequences of *E. coli* cytochrome b_562_ and 4TM are shown. The α-helixes of cytochrome b_562_ and the predicted TM helices of 4TM are delineated by bars. The underlines identify inserted sequences in 4TM used to elongate its TM segments. The residues highlighted in gray are identical in the corresponding sequence positions of both proteins. The position numbers indicate the starting positions of the reporter tags, BlaM and EGFP. (**D**) WB of the *E. coli* C41(ED3) membrane fraction after the expression of D94N-4TM-EGFP (right lane). The left lane shows the membrane fraction of *E. coli* that had been transformed with unmodified pET21-b(+). Protein was visualized by WB with an anti-His antibody against the His-tag of the fusion protein, where the His-tag is located between D94N fusion tag and 4TM.

Previously, we generated D94N to improve the expression levels of some membrane proteins^17^,^22^. Because the C-terminus of D94N is oriented in the membrane as C_in_ (http://opm.phar.umich.edu/)^12^, fusing a TM protein with a naturally occurring N_in_ orientation downstream to D94N should potentially increase its expression. Therefore the increased expression could suggest that the fused TM target protein has a N_in_ orientation. In order to produce the target TM proteins with N_out_ termini, a different D94N-related fusion partner need to be generated. We used a four helical bundle TM protein derived from *E. coli* cytochrome b_562_ as the starting point. We tested this newly designed 4TM and its subsystems of 1TM, 2TM and 3TM as possible linkers. The computational design strategy is described in the Method section (Fig. 1A).

The successful expression of D94N-4TM-EGFP in *E. coli* C41(DE3) was confirmed by Western blotting (WB) using an anti-His antibody (Fig. 1D). A clear band was detected at the anticipated molecular mass of D94N-4TM-EGFP and migrated more rapidly than expected for TM proteins (theoretical molecular mass, 73.4 kDa). At the bottom position a band with the molecular mass corresponding to D94N/His-tag fragment was detected, suggesting the adventitious proteolysis of the fusion protein. The product does not have the red color of heme protein, indicating that 4TM is no longer a heme-binding protein.

To see if the 4TM is able to form a stable conformation, 4TM-EGFP was purified and released from D94N-4TM-EGFP by TEV protease and then characterized by fluorescence-detection size-exclusion chromatography^23^ in the buffer (50 mM Tris-HCl, pH 7.5, 500 mM NaCl, 0.01% n-dodecyl-β-d-maltopyranoside). The result showed that 4TM-EGFP has a very symmetrically peak shape, suggesting a single conformation (Supplementary Fig. S2). Taken together, it appears that 4TM is stable in the *E. coli* plasma membrane, which permits us to test if the odd-helix derivatives of 4TM can be a linker with a C_out_ orientation.

### Verifying that the predicted TM sequences of 4TM are inserted into the membrane

**Figure 2:**
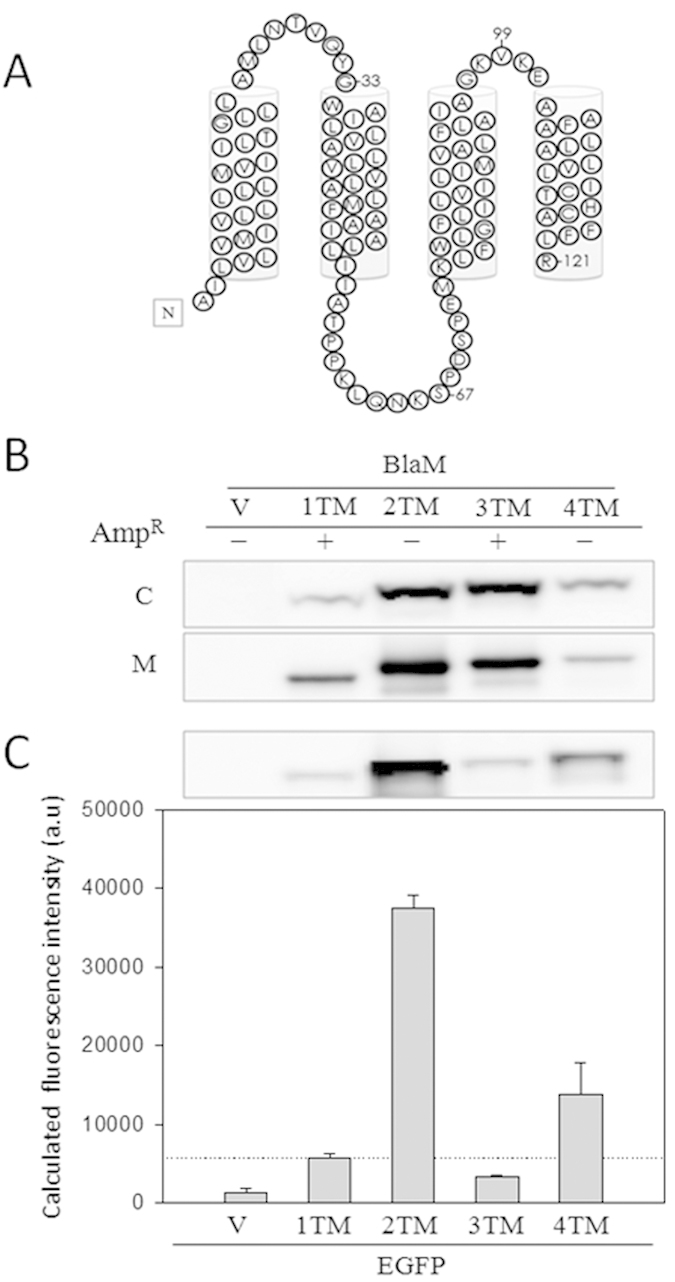
Determination of the 4TM topology in the *E. coli* plasma membrane using reporter tags. (**A**) The predicted topology and sequence of 4TM is shown. The predicted TM segments are represented as cylinders. The position numbers identify the starting positions of the reporter tags, BlaM and EGFP. (**B**) Ampicillin resistance capability of the clones transformed with the BlaM-related constructs is indicated by Amp^R^: +, resistance; −, no resistance. The WBs were used to assess the expression of the BlaM-related constructs in intact cells (**C**) and the membrane fraction (M). V is unmodified pET42-b(+). (**C**) The intact cell WB and relative whole cell fluorescent intensity of the EGFP-related clones are shown. The raw fluorescent intensity of each EGFP-related clone was divided by the cell density (OD_600_). The value was then divided by that of D94N-1TM-EGFP clone to acquire the relative intensity (fold). V is unmodified pET21-b(+). Data are the mean ± S.D. of at least three independent experiments.

To determine whether 4TM is embedded in the membrane and the C-terminal orientation of each TM, we individually fused BlaM or EGFP at R121 of 4TM and derivatives of 4TM that contained one, two, or three predicted TM helices (from the N- to C-termini of 4TM and denoted 1TM, 2TM, and 3TM, respectively; see Fig. 1C and Fig. 2A for the truncation sites). BlaM and EGFP have been routinely used to determine the C-terminal orientations of TM proteins^8^,^9^. BlaM is a periplasmic reporter and protects cells from β-lactam antibiotics only if it translocates to periplasm. Τhe C-terminal codon of each construct was fused to the mature BlaM DNA sequence and cloned into pET-42b(+). To confirm that BlaM was well expressed, folded, and functional, patch tests were performed^24^. All clones were viable at 10 μg/mL ampicillin, indicating that functional BlaM is expressed in each clone (Supplementary Fig. S3A). The relative migration patterns of the fusion proteins detected by WB for whole cells and the membrane fractions presented as a ladder for increasing molecular mass (Fig. 2B), also demonstrating that all constructs are fully and stably expressed. Meanwhile, spot test showed that only clones expressing D94N-1TM-BlaM or D94N-3TM-BlaM formed colonies at 50 and 100 μg/mL ampicillin (Fig. 2B and Supplementary Fig. S3B), whereas the systems expressing D94N-2TM-BlaM and D94N-4TM-BlaM did not. The results suggest that TM1 and TM3 are oriented as N_in_-C_out_ and that TM2 and TM4 are oriented as N_out_-C_in_. The periodic relationship between the four 4TM constructs and antibiotic resistance indicates that 4TM contains four membrane-spanning segments.

To provide additional evidence, we replaced BlaM with the cytosolic reporter, EGFP. Fluorescence of GFP can be detected when GFP localized to cytoplasm, whereas it is inhibited if GFP translocated to periplasm where GFP is improperly folded and degraded^25^. The fluorescence of intact cells that expressed D94N-1TM-EGFP was taken as the cut-off value for its C_out_ orientation deduced by the result from BlaM tag (Fig. 2C). The calculated fluorescence intensities of cells expressing D94N-2TM-EGFP and D94N-4TM-EGFP were above the cut-off value (Fig. 2C). That the whole cell fluorescent intensity of D94N-2TM-EGFP construct is 2.4-fold to that of D94N-4TM-EGFP may come from the net expression levels of them (Fig. 2C). These results are consistent with those of the BlaM-fusion tag experiments in that both types of experiments suggest that TM2 and TM4 have C_in_ orientations and TM1 and TM3 have C_out_ orientations.

Taken together, the results suggested that 4TM is a multi-spanning protein, and it possesses a topology consistent with our predicted model (Fig. 2A). Moreover, 1TM and 3TM linked after D94N can successfully modify the C-terminal orientation to C_out_.

### Development of an expression fusion partner specific for TM proteins with an N_out_ orientation

**Figure 3:**
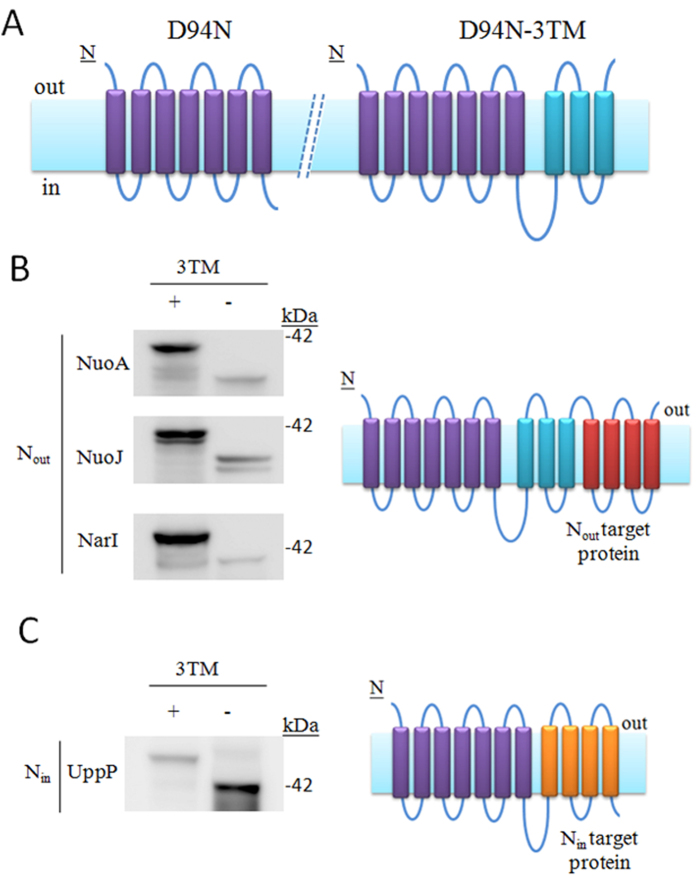
D94N-3TM as a fusion protein for N_out_ TM protein identification and expression. (**A**) The schematics depict the expected orientations of D94N (purple) and D94N-3TM (purple/cyan) in a membrane. (**B**,**C**) The TM proteins were fused downstream to D94N-3TM (+) or D94N (−), expressed, and visualized by WB. Right panel, the schematics illustrate the probable topologies of N_out_ (crimson) and N_in_ (orange) TM proteins when fused to the compatible fusion partner. A integrity topology is expected when the orientations of the targeted TM protein and fusion protein are compatible.

D94N fusion partner has a C_in_ orientation^17^ (Fig. 3A, left), therefore it should only allow for the proper insertion of the TM proteins possessing N_in_ orientations. Notably, the D94N-1TM-BlaM and D94N-3TM-BlaM constructs should have C_out_ orientations allowing BlaM in the periplasm space. Because D94N-3TM-BlaM has a higher net expression level than does D94N-1TM-BlaM (Fig. 2B), we assessed if D94N-3TM could be a possible fusion partner for a N_out_ TM protein (Fig. 3A, right). Even though the designed protein folds as a four helix in the membrane cannot guarantee the 3TM as a stable linker, but a higher net expression of D94N-3TM than that of D94N-4TM indicated that D94N-3TM is also a stable complex. We thus individually fused three N_out_ TM proteins, which are NADH dehydrogenase subunit A (NuoA) and subunit J (NuoJ)^20^, and nitrate reductase 1 gamma subunit (NarI)^21^ from *E. coli* BL21(DE3) to D94N-3TM and D94N and assessed their net expression levels. WB of each expressed DN94N-3TM constructed, e.g., D94N-3TM-NarI, showed a clear band at its anticipated position (Fig. 3B, +), providing evidence that D94N-3TM can be used for expression of TM proteins with an N_out_ orientation.

To validate that the observed levels of NarI, NuoA, and NuoJ expression require D94N-3TM, the expression level of each construct containing only D94N fused upstream of each protein was assessed (Fig. 3B, −). In each case, the WB signal was much weaker when the 3TM sequence was not included in the constructs, indicating that the fusion of only D94N cannot increase the net expression of NarI, NuoA, and NuoJ. Given these results D94N-3TM appears to be a useful fusion partner for N_out_ TM proteins expression. The protein fused downstream to D94N-3TM is functional, as in the case of BlaM (Fig. 2B and Supplementary Fig. S3), further suggesting that D94N-3TM can be applied to protein expression.

The three N_out_ TM proteins, NuoA, NuoJ, and NarI, are better expressed when fused with D94N-3TM than with D94N, likely because the C-terminal of D94N-3TM and the N-terminal of each N_out_ TM protein are both located in the periplasm (Fig. 3B, right panel). To validate this argument, we assessed the expression of UppP from *E. coli*, which has been predicted to have an N_in_ orientation^22^, fused to D94N-3TM or D94N. By visualizing the protein product by WB, it is apparent that expression of UppP is increased when fused to D94N than to D94N-3TM (Fig. 3C), a finding which supports our expectation that UppP has an N_in_ orientation.

By surveying the practicability of D94N-related fusion partners on expression of the four TM proteins, of which the N-terminal orientations have been known and proposed, D94N-3TM fusion partner, as our expectations, can only be applied on the expression of NuoA, NuoJ, and NarI, while D94N fusion partner can only be used to express UppP. Therefore, the D94N-3TM fusion vector should identify N_out_ TM proteins and the D94N fusion vector should identify N_in_ TM proteins. When expressing a target TM protein using both expression fusion partners, its status as an N_in_ or N_out_ could be independently confirmed.

### Extracting N-terminal topological information using the two expression vectors

**Figure 4:**
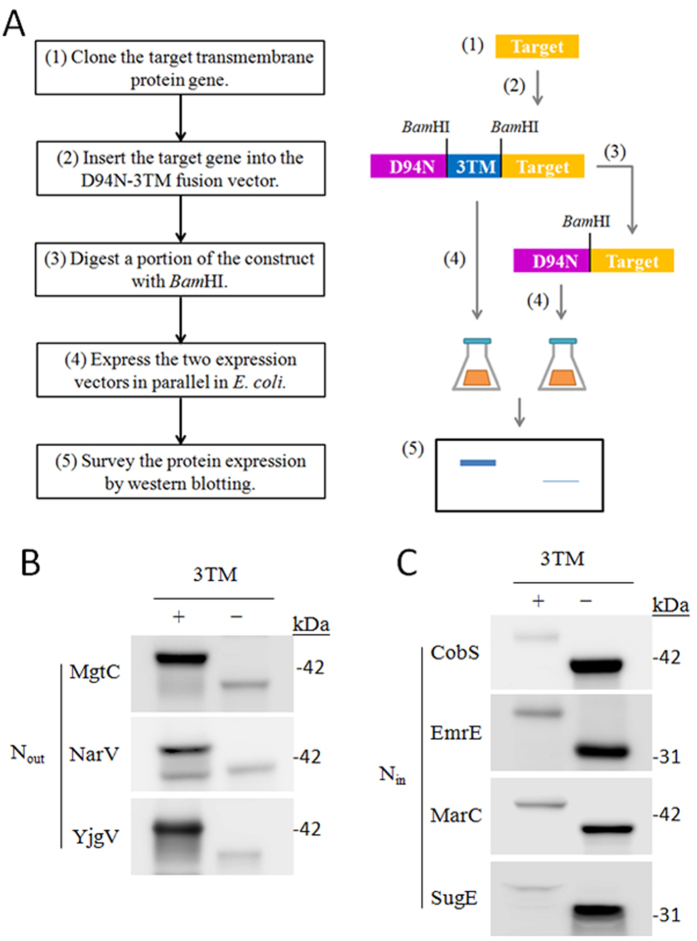
Investigation of the N-terminal orientations of TM proteins using the two–fusion-vector protocol. (**A**) The flowchart and cartoons describe the protocol used to determine the N-terminal orientation of a TM protein. The results from this protocol indicate that the proteins in (**B**) have an N_out_ orientation and those in (**C**) have an N_in_ orientation. The TM proteins were expressed using the D94N-3TM (+) or D94N (-) fusion vector. See Table 1 for the UniProt accession numbers.

We then addressed the orientation issue by examining the compatibility of our expression fusion partners with the expression of various TM proteins. We fused D94N-3TM or D94N with the individual seven additional TM proteins, which are cobalamin synthase (CobS), multidrug efflux protein (EmrE), multiple antibiotic resistance-related protein (MarC), predicted Mg^2+^ transport ATPase (MgtC), respiratory nitrate reductase 2 gamma chain (NarV), multidrug efflux system protein (SugE), conserved inner membrane protein (YgjV) (Fig. 4B,C). These randomly selected TM proteins^13^ were subjected to the protocol diagrammed in Fig. 4A. We also predicted their orientations using TMHMM (http://www.cbs.dtu.dk/services/TMHMM/)^2^, TOPCONS (http://topcons.cbr.su.se/)^26^, and Phobius (http://phobius.sbc.su.se/)^27^ (Table 1). To minimize the number of steps required, the genes were first cloned into the D93N-3TM fusion vector (*see* Supplementary Fig. S4 for the cloning strategy), and then a portion of each preparation was treated with *Bam*HI to remove the 3TM sequence. The *Bam*HI sites are in-frame and the corresponding codons (Gly-Ser) would not be involved in the topological determination. The restriction enzyme cutting sites can be strategically changed if the target genes contain *Bam*HI site(s) during one’s cloning procedure. The N-terminal orientation of each TM protein was assessed by evaluating its expression after insertion into D94N-3TM and D94N fusion vectors by WB analysis, where the fusion proteins were all at the anticipated molecular weight (Fig. 4B,C). Expression of CobS, EmrE, MarC, and SugE is more compatible with D94N fusion vector, which indicates that they have an N_in_ orientation (Fig. 4B). Conversely, expression of MgtC, NarV, and YgjV is better when the D94N-3TM fusion vector is used, suggesting that they possess an N_out_ orientation (Fig. 4C).

We thus try to assess the C-terminal orientation of CobS and MarC fused to D94N and MgtC, NarV, and YgjV fused to D94N-3TM by BlaM and EGFP protein tags (Supplementary Fig. S5). Only the cells expressing D94N-CobS-BlaM showed resistance to 50 μg/mL ampicillin, suggesting the C-terminus orientation of CobS is C_out_. The fluorescent intensity of the cells expressing D94N-CobS-EGFP was used as cutoff value to assign the C-terminus orientation of the other target TM proteins fused with EGFP. The C-termini of D94N-3TM-MgtC, D94N-3TM-NarV, and D94N-3TM-YgjV are accordingly assigned to C_in_ for the significant fluorescent intensities (Supplementary Fig. S5). Taken these together, except MarC, both N- and C-terminal orientations of CobS, MgtC, NarV, YgjV can be obtained and they are N_in_/C_out_, N_out_/C_in_, N_out_/C_in_, and N_out_/C_in_, where the orientations of CobS and MgtC cannot be determined before^13^.

## Discussions

A fundamental question related to TM protein biogenesis still needs to be fully answered. How does a TM protein assume its proper topology and fold into its functional state in the lipid environment? For most membrane proteins, the first hydrophobic segment is responsible for membrane targeting through the translocon and initiates topogenesis at the site^14^,^28^,^29^. It has been difficult to determine the N-terminal orientation of a TM protein experimentally^9^ because the membrane anchor sequence is involved in membrane targeting and the early stage of TM protein biogenesis^1^,^14^,^29^. Bioinformatics software have been introduced to address topogenesis^2^,^4^,^6^,^7^,^26^,^27^. However, these programs sometimes assign different topologies to a given TM protein^7^ (Table 1). Misidentification of the orientation can result in an incorrect prediction model of a TM protein^7^. Furthermore, it is increasingly apparent that TM protein biogenesis and lipid-protein interaction also complicate TM protein topogenesis determinants^5^,^14^, and the orientation of TM proteins is governed by multiple factors^5^,^9^,^29^. Consequently the prediction algorithms may not contain sufficient information. Therefore, it is necessary to discriminate the prediction topology models of a TM protein by experimental data. The strategy^30^,^31^, involving fluorescein 5-maleimide labeled at N-terminus and the reporter proteins fused at C-terminus, had been performed to discriminate the models of secondary transporters generated by prediction algorithms.

In our designed system, by expressing a target TM protein using D94N-3TM and D94N fusion vectors without the need for site-directed mutation and/or chemical labeling, its N-terminal orientation which has been difficult to experimentally determine, can be easily accomplished. GFP may also work at N-terminus, however, fusing GFP at N-terminus of the N_out_ TM proteins can impede its expression^16^. The fusion partners apparently can be a orientation effector for determining the topology of the downstream TM segments. As in the cases of NarI, NuoA, and NuoJ (Fig. 3B), the target TM proteins can only be properly expressed when fused to a topologically compatible fusion partner, i.e., D94N-3TM in the case of NarI. In contrast, a topologically incompatible fusion partner would disturb the topology and, consequently, the folding of the targeted protein. Changing the topological signal of a TM protein may disrupt its topology integrity^15^,^32^, which is a decisive prerequisite for stable TM protein folding in the lipid environment^5^. Quality control of membrane protein expression and folding removes mistranslated, misfolded, unstable, or malfunctioning TM proteins from Gram-negative bacterial inner membranes by a process that involves assessing the stability of the protein via a membrane protease-dependent mechanism^14^,^18^,^19^. Cross-linking and co-purification data have indicated that a membrane-bound protease FtsH involved in quality control forms a complex with a membrane-bound chaperone. This suggests that proteolytic quality control may function during TM protein biogenesis^33^. If a TM protein is fused improperly to D94N-3TM or D94N, the integrity of its topology is probably disturbed, which may subsequently disturb proper folding of the protein in the membrane. A TM protein that does not possess properly oriented TM segments in the membrane cannot be able to form the correct tertiary structure^5^ and may be removed by the quality control system. This scenario may explain the different expression levels of target proteins when fused downstream of D94N-3TM or D94N (Figs 3B,C and 4B,C). In all cases the TM proteins (Figs 3 and 4) were well expressed when fused to the correct expression vector, demonstrating the effectiveness of our method in determining their N-terminal orientation.

EmrE and SugE, which belong to the small multidrug resistance protein family, can present in either orientation in the membrane (dual-topology)^4^,^5^, are included in our study. EmrE is known to form and function in an antiparallel homodimer^34^,^35^,^36^,^37^, but the formation of antiparallel homodimer is still a question. However, EmrE and SugE showed only a N_in_ orientation by our method which might indicate that EmrE and SugE should be initiated from cytoplasm during topogenesis. This result suggests a topological reorientation scenario^4^, i.e., the TM protein first inserts into the membrane in one orientation and then switches to the final state. Seppälä S *et al.*^36^ had demonstrated that the topology of TM proteins remains uncertain till the last reside has been translated. Initiation at the other site, as the case of D94N-3TM-EmrE, might be adverse to topogenesis due to the asymmetry character of lipid bilayer^5^,^38^. Although a cellular system directly involved in topological reorientation has not been found, posttranslational reorientation has been reported for eukaryotic, bacterial, and viral TM proteins^4^,^5^. The bioinformatics analysis indicates that dual orientation of small multidrug resistance proteins may be associated with the lipid composition^39^ which is known to regulate reorientation of TM proteins^5^,^40^,^41^. Perhaps the reorientation of small multidrug resistance proteins is induced by a lipid composition change in membrane microdomains driven by specific cellular conditions, e.g., stress^18^,^38^. Since some data had suggested that the dimerization of small multidrug resistance transporters can stabilize the complex^37^, we cannot exclude the possibility that the instability of D94N-3TM-EmrE and -SugE is due to a possible impeded dimerization with the endogenous EmrE and SugE.

In addition, we could not detect recombinant CobS in *E. coli* system (Supplementary Fig. S6), and, therefore, its orientation would have been impossible to investigate biochemically^13^. However, by fusing CobS to D94N, it was expressed at a detectable level, and its N-terminal orientation was, therefore, easily determined (Fig. 4C). Our D94N-based expression vector had been shown to increase the amounts of some TM proteins expressed in *E. coli*^17^, which is useful when a TM protein is otherwise poorly expressed^22^ and increase the possibility to obtain the C-terminal orientation of the poorly expressed TM proteins, as the case of CobS (Supplementary Fig. S5). In addition, the C-terminal orientation of UppP also determined by the same strategy had been published^22^.

In summary, we present herein an easy method that provides experimental evidence for the orientation of the N-terminal end of a TM protein in a membrane environment, and thereby eliminates the need for predictive algorithms that may not incorporate the complexity needed to accurately determine TM protein topology. We validated our method using three TM proteins with known N_out_ orientations and one protein with a proposed N_in_ orientation^22^ (Fig. 3A,B). We also applied our method to seven other TM proteins (Fig. 4B,C). Combining our method with other biochemical and computational tools will allow a more comprehensive assessment of the topology of TM proteins.

## Methods

### Protein and DNA sequence design

The crystal structure of soluble cytochrome b562 from *E. coli* (PDB code: 3DE9)^42^ was selected as the initial model, while the cytochrome b of membrane-bound cytochrome bc1 complex was selected as the reference for amino acid substitution (PDB code: 3H1J; chain C)^43^. In cytochrome b there are two heme group in the four-helical core (TM1-TM4). The environment of the protruding heme group of cytochrome b is similar to that of cytochrome b562 and used as the base for superimposing their four-helical structures (Fig. 1A). The exterior residues of cytochrome b562 were replaced by the corresponding ones on the cytochrome b (Fig. S1). The residues that cannot be well aligned were changed to the most frequent residues found in TM segments^44^ based on the reasonable interaction of which to milieu. Two helical turns, ILTGLLLAM and VQYGWLI, were captured from the cytochrome b and inserted at the C-terminus of TM1 and the N-terminus of TM2 to achieve the thickness of a membrane (Fig. 1C, underlined; Fig. S1). The structural modeling and refinement was done by PyMol^45^ and the geometry was optimized by the structural idealization function in RefMac^46^. The prediction of ΔG for transmembrane helix insertion was done by the ΔG prediction server (http://dgpred.cbr.su.se/index.php? p = TMpred)^47^ while the calculation of hydrophobic layer was completed by PPM server (http://opm.phar.umich.edu/server.php)^48^. All four helices are predicted to have a negative value of ΔG_app_, which suggests that the sequence is recognized as a TM segment by the Sec translocon. Moreover, the computational result of PPM server (http://opm.phar.umich.edu/server.php)^48^ indicates the depth of the hydrophobic layer is around 32 Å in the model, which agrees with the thickness of a membrane.

### Gene cloning and protein expression

The DNA sequence of 4TM was generated by the “Codon Optimization Tool” at the website of Integrated DNA Technologies Inc. (http://sg.idtdna.com/CodonOpt), which optimizes the DNA sequence for increasing the possibility of the high-yield protein expression in *E. coli*. This sequence was inserted downstream to the gene of D94N/His-tag^17^, and upstream of enhanced green fluorescent protein (EGFP) in the expression vector, pET21-b(+). A stop codon TAA was inserted at the end of EGFP DNA sequence. The DNA sequence was confirmed by using the “Rare Codon Tool” from the website of GeneScript Inc. (http://www.genscript.com), where the codon adaptation index (CAI) was suitable for the *E.coli* (CAI = 0.72). The primer sequences used to clone the genes of target membrane proteins from BL21(DE3) were obtained from the European Nucleotide Archive (http://www.ebi.ac.uk/ena). All needed oligonucleotides were synthesized by Genomics BioSci & Tech (Taiwan). The DNA sequences were cloned from BL21(DE3) genome by PCR and then inserted into D94N-3TM and D94N^17^ fusion vectors. The inserted sequences were confirmed by sequencing. The plasmids were transformed into *E. coli* C41(DE3) and the cells were incubated at 37 °C in Terrific broth^49^ containing 100 μg/mL ampicillin. When OD_600_ of medium reached 0.6, 0.4 mM isopropyl-β-D-thiogalactoside and 5 mM all-*trans* retinal (Sigma) were added and the cells were incubated for another 16 h at 20 °C. After spun down at 4000 × g for 10 min, the cells were resuspended in the buffer, 50 mM Tris-HCl pH 7.5, 500 mM NaCl, benzonase 2 U/ml, lysozyme 200 μg/ml, and protease cocktail (Roche). The cells were disrupted by Constant Cell Disruption Systems (Constant Systems Ltd) and centrifuged at 4000 × g for 10 min. Each supernatant was centrifuged at 84,000 × *g* for 35 min to harvest the membrane fraction. The precipitates were washed with 10-fold volumes of storage buffer, 50 mM Tris-HCl pH 7.5, 500 mM NaCl, 10% (w/v) glycerol, before centrifugation at 84,000 × *g* for 1 h. Finally, the membrane fraction was resuspended in the storage buffer and stored at −80 °C for later use. Protein concentrations were measured by the Bio-Rad Protein Assay.

### Western blotting analysis

The total protein of 50 μL induced cell culture (OD_600_ of 1.0) or 20 μg membrane fraction was resolved on 12% SDS-PAGE. The protein was transferred onto nitrocellulose membrane and visualized by following the standard protocol of SuperSignal^TM^ West HisProbe Kit (Thermo Fisher Scientific).

### Spot test and patch test

By the method described in the article^24^ with minor modifications, the transformed cells were firstly incubated in Luria-Bertani broth containing 50 μg/mL kanamycin till OD_600_ was 1.0 and then induced by 0.4 mM isopropyl-thio-β-D-galactopyranoside for 5 h at 37 °C. For spot assay, the culture mediums were diluted 1000-fold and 1 μl of the dilutions was spread onto the culture plate containing 50 and 100 μg/mL ampicillin and 0.2 mM isopropyl-thio-β-D-galactopyranoside. For patch assay, 1μl of each cultured medium was spread onto the culture plate containing 10 μg/mL ampicillin and 0.2 mM isopropyl-thio-β-D-galactopyranoside. The plates were incubated for 16 h at 37 °C to see the consequences of colony growth.

### Whole cell fluorescence

The whole cell fluorescence estimation followed the protocol described by Daley *et al.*^13^. After washed by the buffer containing 50 mM Tris-HCl pH 8.0, 200 mM NaCl, and 15 mM EDTA, the cell pellet was resuspended in the same buffer and incubated at room temperature for 1h, and assayed for EGFP fluorescence. Fluorescence was measured in Synergy™ H4 Hybrid Multi-Mode Microplate Reader (BioTEK) with an excitation wavelength of 485 nm and an emission wavelength of 520 nm.

## Additional Information

**How to cite this article**: Lee, C.-H. *et al.* Determining the N-terminal orientations of recombinant transmembrane proteins in the *Escherichia coli* plasma membrane. *Sci. Rep.* **5**, 15086; doi: 10.1038/srep15086 (2015).

## Supplementary Material

Supplementary Information

## Figures and Tables

**Table 1:** Topology results from prediction by TMHMM^2^, TOPCONS^26^, and Phobius^27^, and the experimental data.

	AminoAcidNumber	TMHMM	TOPCONS	Phobius	ExperimentalResult	Reference^a^
CobS	247	Out/Out/4	In/Out/7	In/In/6	In/Out^b^	
EmrE	110	Out/Out/4	Out/Out/4	Out/In/3	Dual-topology	(13)
MarC	221	Out/In/5	Out/Out/6	Out/In/5	In/In	(13)
MgtC	215	Out/In/5	Out/Out/6	Out/In/5	Out/In^b^	
NarI	225	Out/In/5	Out/In/5	Out/In/5	Out/In^b^	(21)
NarV	226	Out/In/5	Out/In/5	Out/In/5	Out/In^b^	(13)
NuoA	147	Out/In/3	Out/In/3	Out/In/3	Out/In	(20)
NuoJ	184	Out/In/5	Out/In/5	Out/Out/4	Out/In	(20)
SugE	105	In/In/4	In/In/4	Out/In/3	Dual-topology	(13)
UppP	273	In/Out/7	In/Out/7	Out/In/7	In/In	(22)
YgjV	183	In/In/4	Out/In/7	Out/In/5	Out/In^b^	(13)

The UniProt (http://www.uniprot.org/) accession numbers of the target TM proteins are: CobS, P36561; EmrE, C6EKS5; MarC, C6EDW0; MgtC, C6EEH7; NarI, C6EGN6; NarV, P0AF32; NuoA, C6E9R4; NuoJ, C6E9S2; SugE, C6ECX5; UppP, C6EHT8; YgjV, C6EHQ4. The protein sequences applied for prediction were obtained from Uniprot and the results are shown in the order, N-terminal orientation/C-terminal orientation/the number of TMs.

^a^the reference of C-terminal orientation determined.

^b^The C-terminus orientation of the target TM protein assessed in this article.
