# Supplementary material for: Determining the N-terminal orientations of recombinant transmembrane proteins in the Escherichia coli plasma membrane
Source: Sci Rep. 2015 Oct 14;5:15086. doi: 10.1038/srep15086 (PMC4604451; doi:10.1038/srep15086)
Supplement: Supplementary Information [file srep15086-s1.pdf]

Determining the N-terminal orientations of recombinant transmembrane proteins in the *Escherichia coli* plasma membrane

Chien-Hsien Lee[1,2], Chia-Cheng Chou[1,2], Min-Feng Hsu[1,2] and Andrew H.-J. Wang[1,2]

[1]The Institute of Biological Chemistry, Academia Sinica, Taipei, Taiwan
[2]Core Facilities for Protein Structural Analysis, Academia Sinica, Taipei, Taiwan

Figure S1

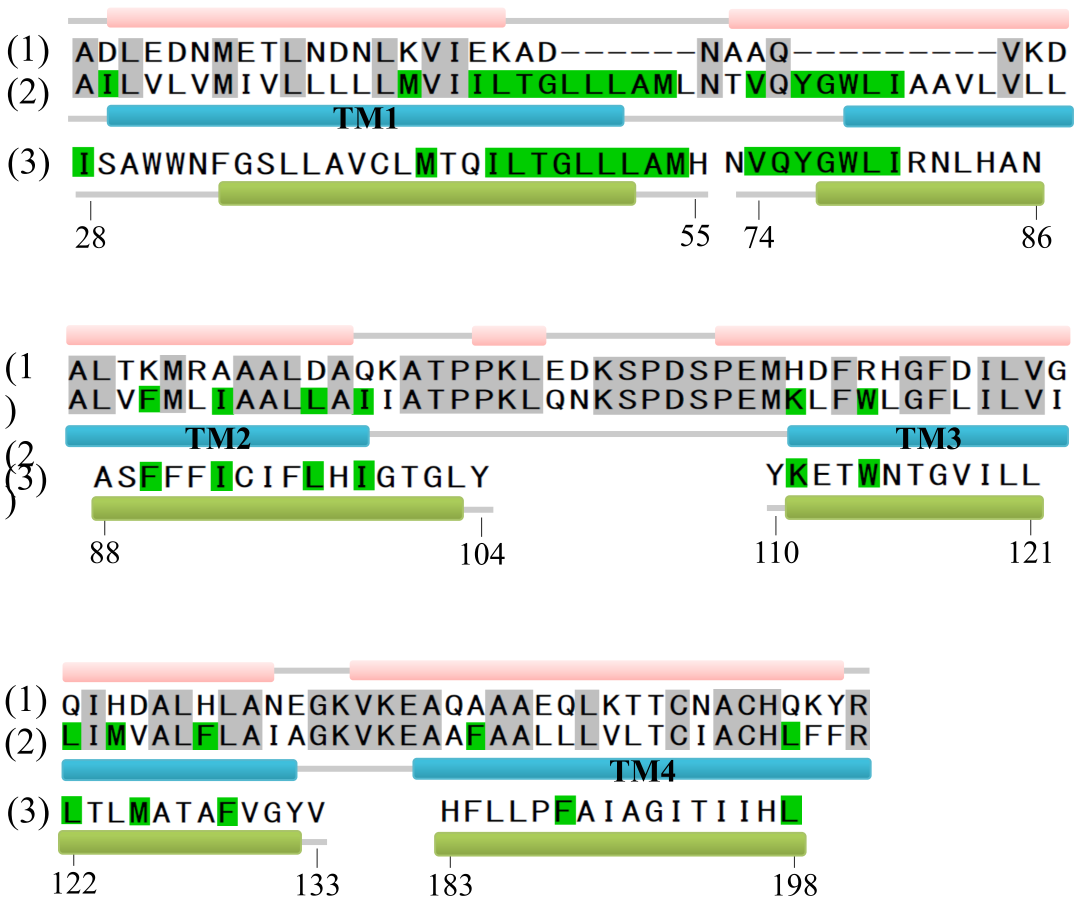

(1) : Mature E. coli cytochrome b562
(2) : designed 4TM
(3) : the four-helix core of cytochrome b

Figure S1. The amino acid sequences of *E. coli* cytochrome b562, designed 4TM, and the four-helix core of cytochrome b form cytochrome bc1 complex are shown. The a-helixes of cytochrome b562 and cytochrome b and the predicted TM helices of 4TM are delineated by bars. The green-shadowed amino acids of 4TM indicate the corresponding ones on chytochrome b. The position number of the amino acid on cytochrome b are shown below.

Figure S2

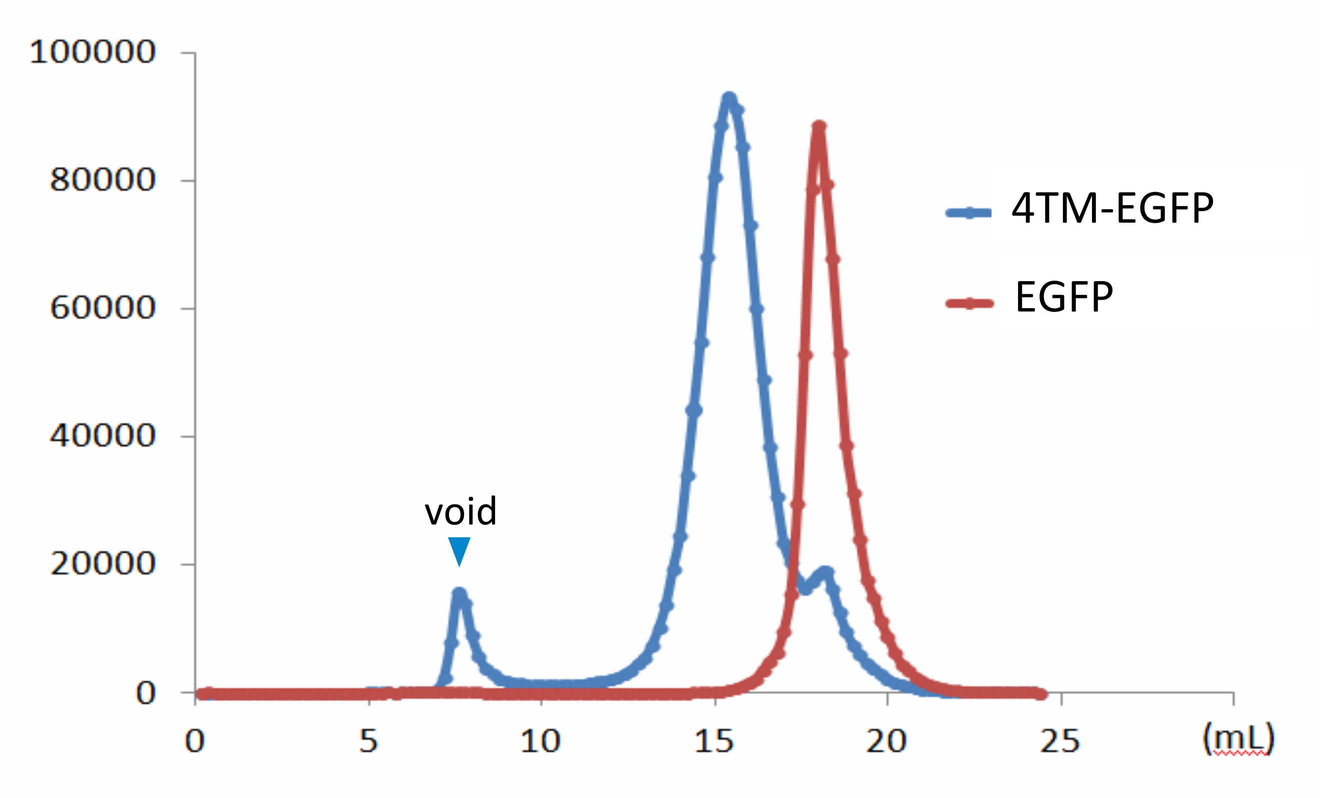

Figure S2. The Fluorescence-Detection Size-Exclusion Chromatography of 4TM-EGFP. The 4TM-EGFP fragment of D94N-4TM-EGFP containing A206→K mutation of EGFP was purified. 4TM-EGFP and EGFP were solubilized in 50 mM Tris-HCl, pH 7.5, 500 mM NaCl, 0.01% n-dodecyl-β-D-maltopyranoside and subjected into superose™ 6 10/300 GL respectively. The fluorescence intensities of each 200 μL fraction of 4TM-EGFP and EGFP were estimated sequentially.

Figure S3

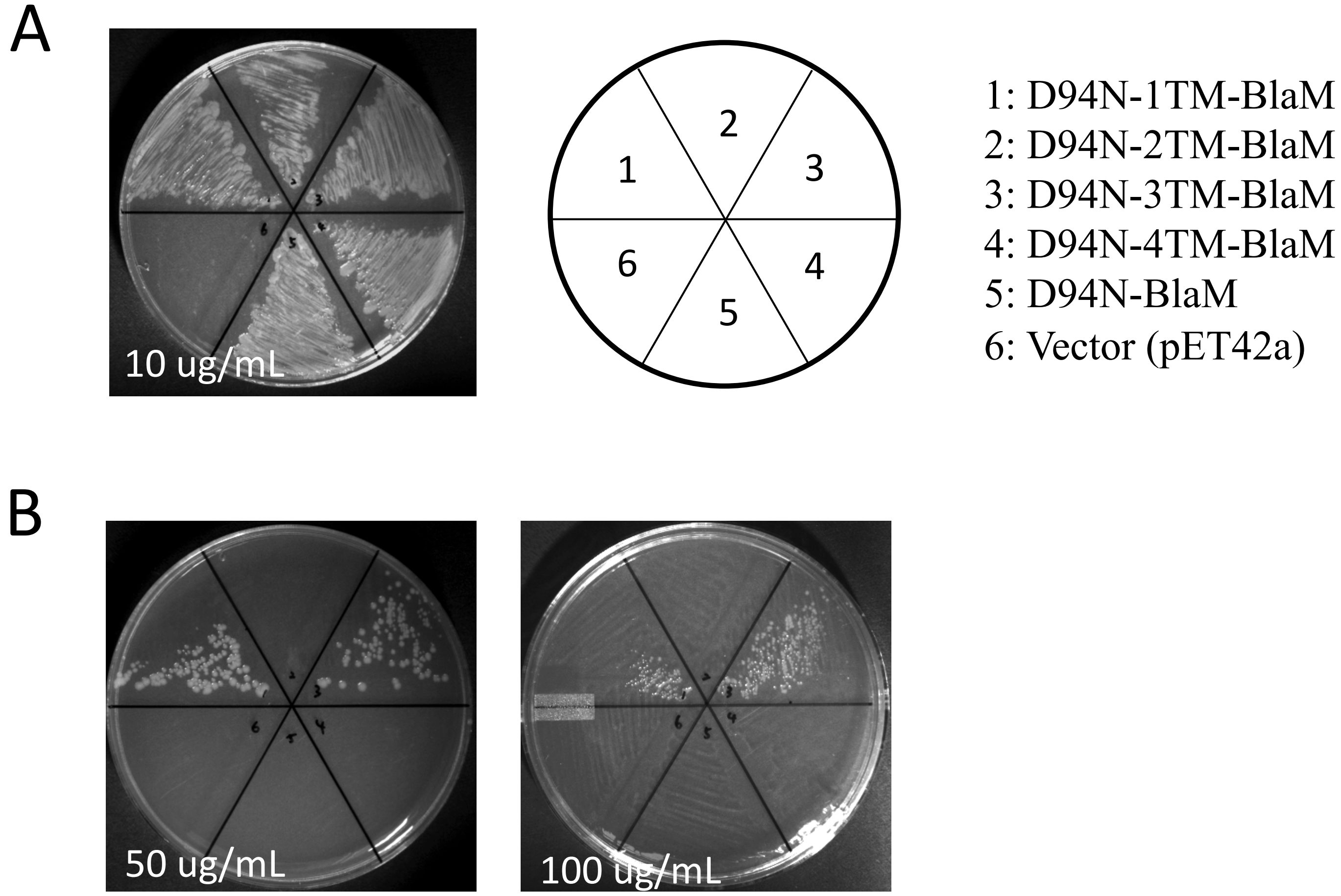

Figure S3. Ampicillin resistance of the cells by (A) patch and (B) spot test (see the main text for the detail). The *E. coli* C41(DE3) was transformed with the construct indicated. The induced cells were spread onto the plate containing 0.2 mM isopropyl-thio-β-D-galactopyranoside and ampicillin (the concentration indicated below). The plate was incubated at 37 °C overnight to assess the ampicillin resistance of the cells.

Figure S4

```
D94N  ggatccgcgatcctggtgctggttatgattgtttactgttgttacttatggttattatt
      G  S  A  I  L  V  L  V  M  I  V  L  L  L  L  L  M  V  I  I

      ctgaccggtcttctcctggcgatgctgaatactgttcagtatggttggctcatcgcagcc
      L  T  G  L  L  L  A  M  L  N  T  V  Q  Y  G  W  L  I  A  A

      gtactggtgctgttggccctggtcttcatgctgatcgcggcactgctggcgattatcgct
      V  L  V  L  L  A  L  V  F  M  L  I  A  A  L  L  A  I  I  A

      actccgccgaaattgcagaacaaatccccggacagtccagaaatgaaactgttctggctt
      T  P  P  K  L  Q  N  K  S  P  D  S  P  E  M  K  L  F  W  L

      ggattcctgattcttgtgatcctgatcatggtcgccctgttttggccattgcaggcaag
      G  F  L  I  L  V  I  L  I  M  V  A  L  F  L  A  I  A  G  K

      gttggatccggt   Target proteins
      V  G  S  G
```

Figure S4. The DNA sequence and amino acid of 3TM linker are shown. The dotted arrows indicate the primer region. BamHI site are shown in red.

Figure S5

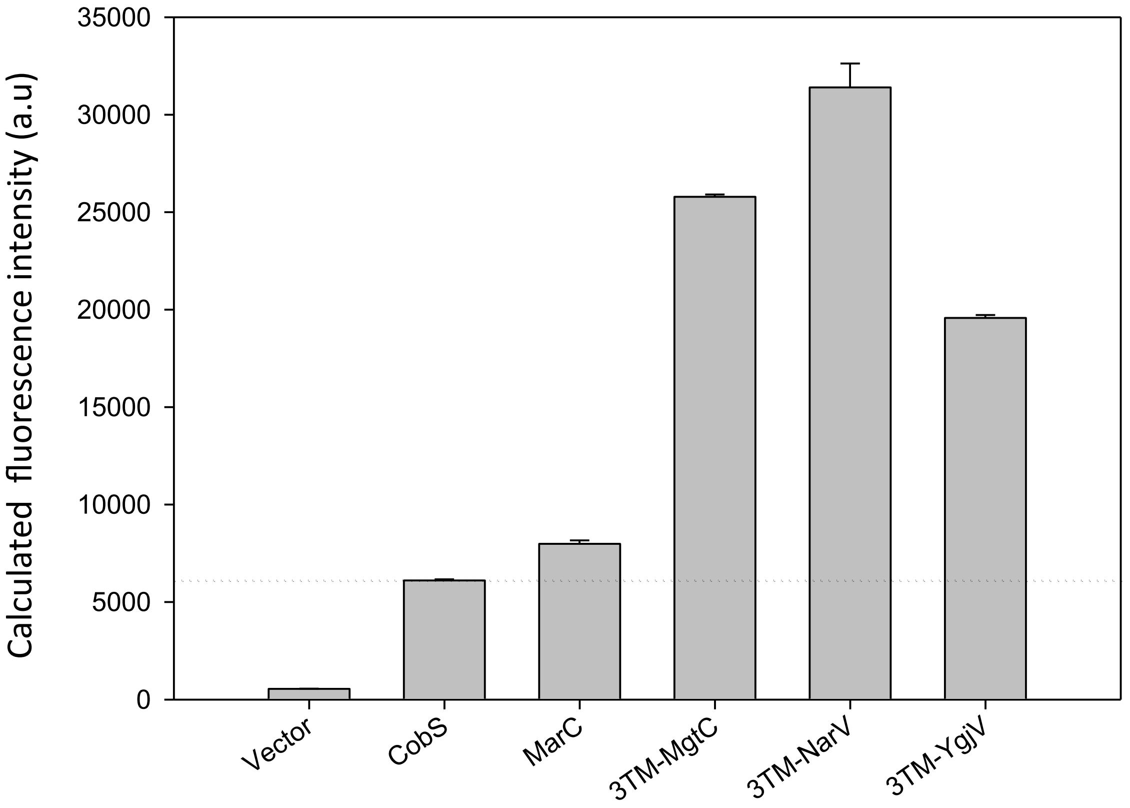

10 µg/mL Amp	–	+	+	+	+	+
50 µg/mL Amp	–	+	–	–	–	–
C-terminus orientation	**	C_{out}	**	C_{in}	C_{in}	C_{in}

Figure S5. The C-terminal orientation assignments of the target TM proteins. The target TM proteins indicated were fused with D94N fusion tag and with either EGFP or BlaM at their C-terminus. The lower panel shows the results of the cells expressing the BlaM-related fusion protein, which was indicated by "+", resistance; "–", no resistance. The upper panel shows the fluorescent intensity of the cells expressing EGFP-related fusion protein. The intensity of D94N-CobS-EGFP was as cut-off value to determine C-terminal orientation of the other target TM proteins. Vector, unmodified expression plasmid. **, can not be assigned.

Figure S6

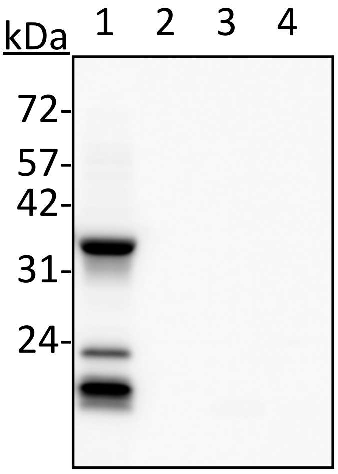

1. D94N-CobS
2. H_6-CobS
3. H_6TEV-CobS
4. CobS-H_6

Figure S4. The expression results of recombinant cobalamin synthase (CobS). CobS was cloned from BL21(DE3) by PCR and fused to D94N or tagged with His-tag. Each construct was transformed into C41(DE3). After the cells induced by 0.4 mM isopropyl-thio-β-D-galactopyranoside for 16 h at 20 °C, the membrane fractions of the cells were extracted. The expression of the recombinant proteins was assessed by western blotting via SuperSignal West HisProbe Kit (Thermo Fisher Scientific). H_6, His-tag; TEV, recognition amino sequence of TEV protease. The theoretical molecular mass for D94N-CobS, 56.7 kDa; H_6-CobS and CobS-H_6, 27.2 kDa; H_6TEV-CobS, 28.1 kDa.

Table S1 The prediction result of TM insertion.

**Range (position number)**	**Length (residue)**	**ΔG_{app} (kcal/ mol)**
2-24	23	-6.330
34-56	23	-5.050
74-96	23	-6.024
102-121	20	-2.698

The four regions of 4TM were analyzed by the prediction server for TM helix insertion (http://dgpred.cbr.su.se/index.php?p=TMpred).
